# Oita virus rediscovered after 50 years: isolation of genetically conserved strains from bats in Southern Japan

**DOI:** 10.1128/spectrum.03158-25

**Published:** 2025-11-11

**Authors:** Saiko Sawai, Kittiya Intaruck, Sho Sata, Mana Esaki, Kimitake Funakoshi, Shin Murakami, Keita Matsuno, Ryo Nakao, Kazunori Kimitsuki, Takaaki Yahiro, Akira Nishizono, Naganori Nao, Yasuko Orba, Ayato Takada, Masahiro Kajihara, Makoto Ozawa, Kosuke Okuya

**Affiliations:** 1 Joint Faculty of Veterinary Medicine, Kagoshima University 199253 , Kagoshima, Japan; 2 Division of International Research Promotion, International Institute for Zoonosis Control, Hokkaido University 12810, Sapporo, Japan; 3 Joint Graduate School of Veterinary Medicine, Kagoshima University 12851, Kagoshima, Japan; 4 Biological Laboratory, Faculty of Intercultural Studies, The International University of Kagoshima 12831, Kagoshima, Japan; 5 Laboratory of Veterinary Microbiology, Graduate School of Agricultural and Life Sciences, The University of Tokyo, Tokyo, Japan; 6 Division of Risk Analysis and Management, Hokkaido University International Institute for Zoonosis Control, Sapporo, Japan; 7 One Health Research Center, Hokkaido University 12810, Sapporo, Japan; 8 Institute for Vaccine Research and Development, HU-IVReD, Hokkaido University, Sapporo, Japan; 9 International Collaboration Unit, International Institute for Zoonosis Control, Hokkaido University 12810, Sapporo, Japan; 10 Laboratory of Parasitology, Graduate School of Infectious Diseases, Faculty of Veterinary Medicine, Hokkaido University 12810, Sapporo, Japan; 11 Division of Parasitology, Veterinary Research Unit, International Institute for Zoonosis Control, Hokkaido University 12810, Sapporo, Japan; 12 Research Center for Global and Local Infectious Diseases, Oita University 154361, Yufu, Japan; 13 Department of Microbiology, Faculty of Medicine, Oita University 13235, Yufu, Japan; 14 Department of Advanced Medical Sciences, Faculty of Medicine, Oita University 13235, Yufu, Japan; 15 Hokudai Center for Zoonosis Control in Zambia, School of Veterinary Medicine, University of Zambia 247512 , Lusaka, Zambia; 16 Division of Molecular Pathobiology, International Institute for Zoonosis Control, Hokkaido University 12810, Sapporo, Japan; 17 Division of Global Epidemiology, International Institute for Zoonosis Control, Hokkaido University 12810, Sapporo, Japan; Barnard College, Columbia University, New York, New York, USA

**Keywords:** rhabdovirus, bat, re-emerging diseases, ledantevirus

## Abstract

Bats are natural reservoirs of numerous viruses, including members of the *Rhabdoviridae* family. Oita virus (OITV), classified within the genus *Ledantevirus*, was first isolated from the blood of a Japanese horseshoe bat (*Rhinolophus cornutus*) in the Oita Prefecture, Japan, in 1972. However, since then, no other OITV isolates have been reported. In this study, we isolated two OITV strains—OITV 321/2022 and OITV 326/2022—from oral swab samples of *R. cornutus* collected in the Katano Cave, Kagoshima Prefecture, Japan. Genetic analysis revealed >98% nucleotide identity with the previous isolate, OITV 296/1972. Phylogenetic analysis confirmed their classification within the *Ledantevirus* subgroup C. We assessed viral growth kinetics using 17 cell lines from diverse animal species, including bats, rodents, humans, and arthropods. OITV 321/2022 showed efficient replication in primate-derived, bat-derived, and human-derived cells, but not in tick or mosquito cell lines. Experimental infection of BALB/c mice demonstrated that OITV 321/2022 induced no overt clinical signs via either the intracerebral or intranasal routes. However, systemic infection and viral replication in the brain and lungs were observed by intracerebral inoculation. Intranasal inoculation with OITV 321/2022 and OITV 296/1972 resulted in transient pulmonary infection without systemic dissemination. This study provides evidence of OITV circulation in wild bat populations over 50 years, highlighting the need for continued surveillance of bat-associated rhabdoviruses in Japan.

**IMPORTANCE:**

Bats are known reservoirs of zoonotic viruses, and their proximity to humans raises concerns regarding zoonotic risks. We report the first isolation of the Oita virus (OITV), a bat-associated ledantevirus, that has circulated for over 50 years. Unlike a previous blood-derived isolate, our isolates were obtained from oral swabs, suggesting their potential for respiratory transmission. OITV could infect a wide range of mammalian cells, including human-derived cells, and induce systemic infection in mice without clinical symptoms. These findings indicate that OITV possesses a broad host tropism and may circulate among microbats through the respiratory tract. Although the pathogenicity of the newly isolated strain appears to be attenuated compared with that of a historical brain-passaged strain, its ability to replicate in human cells underscores its potential zoonotic relevance, necessitating active surveillance and functional characterization of bat-derived rhabdoviruses to better assess emerging infectious disease threats.

## INTRODUCTION

Bats are natural reservoirs of various viruses, including coronaviruses and rhabdoviruses (1, 2). To date, more than 200 viruses have been detected in or isolated from bats, many with the potential of zoonotic transmission (3). Their ability to fly enables long-distance dispersal of viruses, contributing to the widespread geographic distribution of bat-associated pathogens (4). In addition, the formation of large colonies facilitates viral maintenance and intra-species transmission within bat populations (5). Bats are also capable of adapting to urban environments, thereby increasing the frequency of contact with humans and domestic animals and raising the risk of cross-species transmission.

The *Rhabdoviridae* family comprises single-stranded negative-sense RNA viruses with five genes: nucleoprotein (N), phosphoprotein (P), matrix protein (M), glycoprotein (G), and viral RNA polymerase (L) (6). This family exhibits high ecological diversity, with members infecting various hosts, including plants, mammals, birds, reptiles, insects, and fish (7–10). Some genera within the family include important animal and human viruses, such as lyssaviruses (e.g., rabies virus) and vesiculoviruses (e.g., Chandipura virus). An increasing number of novel bat-associated rhabdoviruses have recently been identified in different regions worldwide, highlighting the growing importance of active surveillance of bats as viral reservoirs (6).

The genus *Ledantevirus*, as listed by the International Committee on Taxonomy of Viruses (https://ictv.global/) in 2024, includes 21 distinct viral species. Phylogenetic analyses have subdivided the genus *Ledantevirus* into three major subgroups: A, B, and C (10). Many members of this genus have been isolated from bats and are thought to be transmitted by arthropods (10). Among the ectoparasites of bats, bat flies—obligate hematophagous arthropods—are of particular interest. Kanyawara virus and Bughendera virus have been directly detected in bat flies collected from parasitized bats (11). Additionally, the Kolente virus was isolated from both bats and ticks in the Republic of Guinea in 1985 (12). Furthermore, some ledanteviruses are suspected to be associated with human diseases (6, 13–15). The first identified virus, Ledantec virus, was isolated in 1965 from the serum of a 10-year-old girl with fever and hepatosplenomegaly at Le Dantec University Hospital in Senegal (15). In China, antibodies specific to a novel ledantevirus, Rhinolophus rhabdovirus DPuer (DPRV), were detected in human serum samples collected from individuals living near bat sampling sites who had experienced fever in the past year (6).

The Oita virus (OITV), classified within the genus *Ledantevirus*, was first isolated in 1972 and designated as OITV 296/1972 (16). The virus was obtained from the blood of a small Japanese horseshoe bat (*Rhinolophus cornutus*) captured in Oita Prefecture, Japan. OITV 296/1972 was isolated by intracerebral inoculation into suckling mice, which subsequently developed lethal encephalitis, indicating neurotropism similar to that of rabies virus (16). However, the replication characteristics and growth kinetics of this virus have not been evaluated in cultured cells. As OITV has not been isolated for over 50 years since 1972, its ecology remains largely unknown.

In this study, we isolated OITV from oral swab samples collected from *R. cornutus* captured in Kagoshima Prefecture, Japan. We genetically characterized the OITV and virologically assessed its growth kinetics in a range of cell lines. In addition, we investigated the pathogenicity of OITV in mice.

## RESULTS

### OITV isolation from microbats in Kagoshima Prefecture

**Fig 1:**
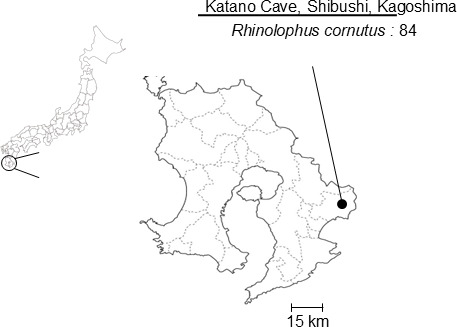
Location of Katano Cave, where microbats were captured in this study. Katano Cave is shown on a map of Kagoshima Prefecture, Japan.

**Fig 2:**
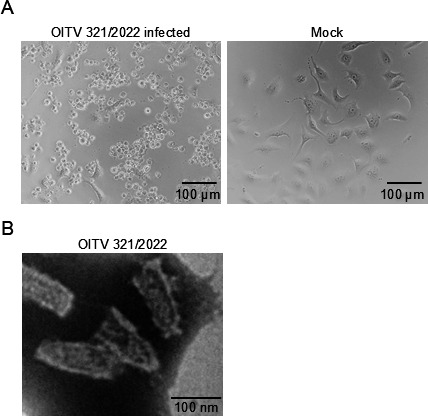
OITV isolation from *R. cornutus*. Cell detachment was observed in Vero-RcACE2 cells inoculated with OITV 321/2022 (**A**). Virus particles were detected in the supernatants of cells inoculated with OITV 321/2022 using transmission electron microscopy (**B**).

Eighty-four oral swab samples were collected from *R. cornutus* at Katano Cave, Kagoshima, Japan (Fig. 1) and inoculated subsequently into Vero-RcACE2 cells. Cytopathic effects (CPE), characterized by cell detachment, were observed 4 days after blind passage in cells inoculated with two swab samples, Bat321/2022 and Bat326/2022 (Table 1; Fig. 2A). Furthermore, bullet-shaped virus particles, characteristic of rhabdoviruses, were observed in the supernatant of Bat321/2022-inoculated cells by transmission electron microscopy (Fig. 2B).

**TABLE 1:** Bat samples that showed CPE

Collection date	Bat ID	Bat species	Sex	Young/adult
27 October 2022	Bat321	*Rhinolophus cornutus*	Female	Adult
	Bat326		Male	Young

Full-genome sequencing revealed that these two virus isolates belonged to the genus *Ledantevirus,* family *Rhabdoviridae*, and were tentatively designated OITV 321/2022 and OITV 326/2022.

### Genetic analyses of OITV 321/2022 and OITV 326/2022

**Fig 3:**
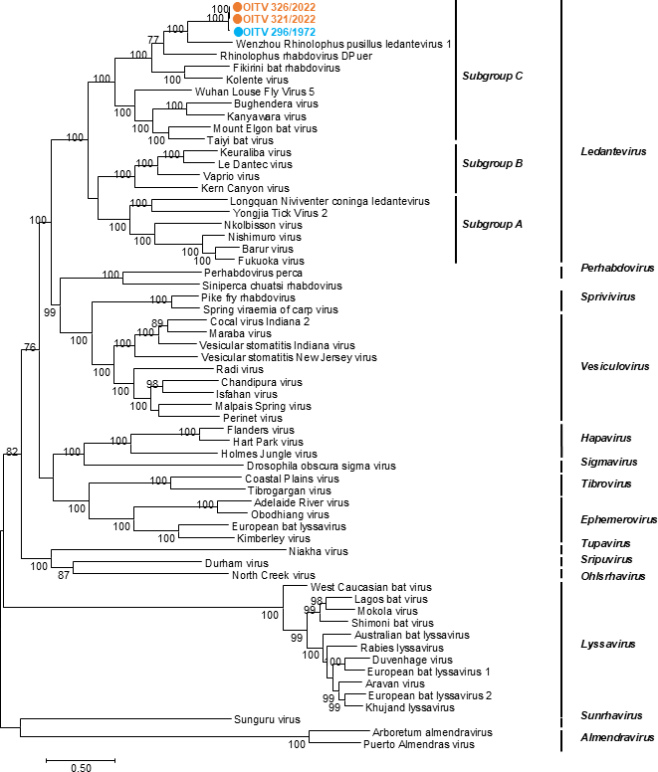
Phylogenetic analyses of *Rhabdoviridae*. Phylogenetic analyses were conducted based on the full-genome nucleotide sequences of the rhabdoviruses. OITV 321/2022 and OITV 326/2022 are represented as orange circles, and OITV 296/1972 as a blue circle.

Genetic analyses revealed that the Kagoshima isolates, OITV 321/2022 and OITV 326/2022, shared a high nucleotide sequence identity (98.52%) with OITV 296/1972, which was isolated from *R. cornutus* in Oita Prefecture, Japan, in 1972 (16). Each viral gene exhibited >97.78% sequence identity between Kagoshima isolates and OITV 296/1972 (Table 2). Phylogenetic analyses further confirmed that OITV 321/2022 and OITV 326/2022 are classified into subgroup C of the genus *Ledantevirus* (Fig. 3) (10). Since the full-length genome sequences of OITV 321/2022 and OITV 326/2022 were identical, OITV 321/2022 was used as a representative strain for subsequent virological characterization in this study.

**TABLE 2:** Nucleotide and amino acid identities between Kagoshima isolates and OITV 296/1972

ORF	Length (bp)	Identity (%)	
		Nucleotide level	Amino acid level
N	1,293	99.15	99.77
P	1,125	97.78	96.79
M	627	98.88	100
G	1,656	98.73	99.09
L	6,360	98.43	99.62

### Cell susceptibility to OITV infection

To evaluate the host range and cell susceptibility to OITV, we first assessed the replication capacity of OITV 321/2022 in a panel of cell lines commonly used in *in vitro* studies. A total of eight cell lines derived from various animal species were tested, including those from nonhuman primates (African green monkeys: Vero, Vero-RcACE2, and VeroE6/TMPRSS2 cells), humans (A549 cells), bats (TB1 Lu cells), hamsters (baby hamster kidney [BHK] cells), dogs (Madin-Darby canine kidney [MDCK] cells), and pigs (PK-15 cells).

OITV 321/2022 replicated in Vero, Vero-RcACE2, and VeroE6/TMPRSS2 cells, with apparent cell detachment at 4–5 days post-infection (dpi). Although no CPE was observed in the remaining cell lines, real-time RT-PCR confirmed viral replication in A549, BHK, and MDCK cells. In contrast, OITV 321/2022 did not replicate in TB1 Lu and PK-15 cells, indicating variable susceptibility to OITV among cell lines derived from different animal species.

### Replication of OITV 321/2022

**Fig 4:**
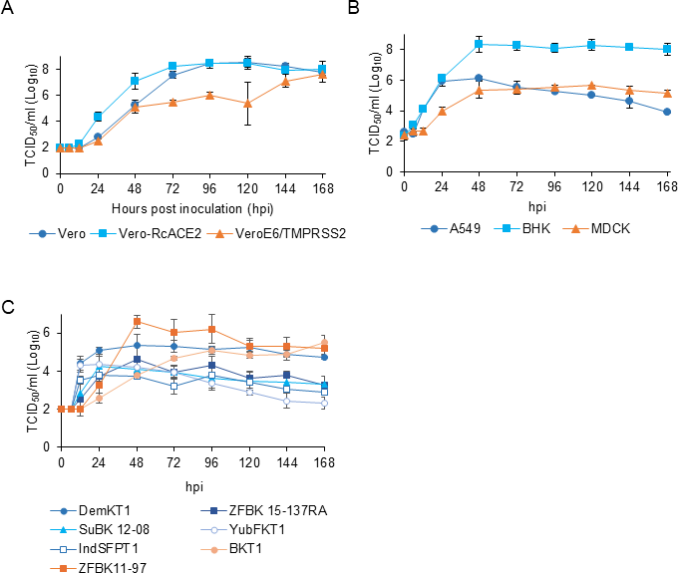
Growth kinetics of OITV 321/2022 in cultured cells. Virus titers of OITV 321/2022 were measured at multiple time points using the TCID_50_ assay with Vero-RcACE2 cells. Growth kinetics were evaluated in CPE-positive (Vero, Vero-RcACE2, and VeroE6/TMPRSS2 cells) (**A**), CPE-negative (A549, BHK, and MDCK cells) (**B**), and bat-derived (DemKT1, ZFBK 15-137RA, SuBK 12-08, YubFKT1, IndSFPT1, BKT1, and ZFBK 11-97 cells) cell lines (**C**).

To investigate the growth kinetics of OITV, OITV 321/2022 was inoculated into six cell lines that supported viral replication (Fig. 4A and B). Culture supernatants were collected at multiple time points and subjected to virus titration using the tissue culture infectious dose (TCID_50_) assay in Vero-RcACE2 cells.

In Vero and Vero-RcACE2 cells, both of which exhibited cell detachment following infection, the viral titers began increasing at 24 hours post-infection (hpi), plateauing at 96 hpi (Fig. 4A). In VeroE6/TMPRSS2 cells, the viral titers gradually increased and reached levels comparable to those observed in Vero and Vero-RcACE2 cells at 168 hpi (Fig. 4A). In contrast, A549, BHK, and MDCK cells did not show CPE but supported viral replication. In these cells, the titers increased from 6 to 12 hpi and plateaued between 48 and 72 hpi (Fig. 4B). Notably, BHK cells supported viral replication at levels similar to those in CPE-positive cell lines, despite no visible CPE.

Ledanteviruses have been detected in bats and are suspected to be associated with arthropod vectors (16). We further tested OITV replication in various cell lines derived from bats, ticks, and mosquitoes (Fig. 4C). Seven bat-derived cell lines from different species were examined for viral replication and growth kinetics. Among bat-derived cell lines, CPE was observed in ZFBK 11-97 and SuBK 12-08 cells at 4 dpi (Fig. S1). Viral titers began to increase at 12 hpi and plateaued at 48 hpi in DemKT1, ZFBK 15-137RA, SuBK 12-08, YubFKT1, and IndFSPT1 cells (Fig. 4C). In contrast, BKT1 and ZFBK 11-97 cells exhibited delayed viral growth, with titers increasing from 24 hpi. Notably, ZFBK 11-97 cells showed the highest viral titers among all bat cell lines, peaking at 48 hpi, while BKT1 cells displayed a gradual increase, reaching a plateau at 96 hpi (Fig. 4C). Overall, OITV 321/2022 replicated most efficiently in ZFBK 11-97 cells among the seven bat cell lines tested. In contrast, viral titers in arthropod-derived cell lines (ISE-6 and C6/36) remained below the detection limit throughout the observation period, suggesting that these cells do not support OITV 321/2022 replication.

### Infectivity of OITV 321/2022 in mice

**Fig 5:**
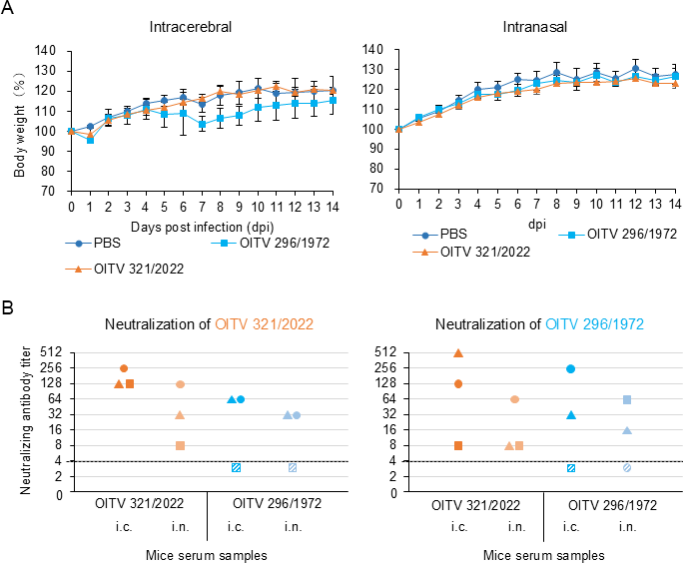
OITV 321/2022 infectivity in mice. BALB/c mice were inoculated intracerebrally or intranasally with either OITV 321/2022 or OITV 296/1972. PBS was administered to the control group. Body weight and clinical signs were monitored daily for 14 days post-inoculation (**A**). Mice were euthanized at 14 dpi, and serum samples were collected for microneutralization assays (**B**). Each symbol represents the antibody titer of an individual mouse, with matching symbols on the *x*-axis corresponding to the same animal. The black dashed lines indicate values below the detection limit.

To assess the pathogenicity of OITV 321/2022, BALB/c mice were inoculated with this strain, using OITV 296/1972 (16) as a positive control. Mice inoculated intracerebrally with OITV 296/1972 exhibited weight loss, whereas those inoculated intranasally exhibited no clinical symptoms (Fig. 5A). In contrast, mice inoculated with OITV 321/2022 via either route showed no clinical signs or significant weight loss (Fig. 5A). Despite the absence of symptoms, serum samples collected on 14 dpi from mice inoculated with either OITV 296/1972 or OITV 321/2022 showed elevated titers of neutralizing antibody, except for one intracerebrally inoculated mouse in the OITV 296/1972 group (Fig. 5B). Furthermore, OITV-specific antibodies were detected by enzyme-linked immunosorbent assay (ELISA) in serum samples from all virus-inoculated mice (Fig. S2), indicating that OITV infection was established via both the intracerebral and intranasal routes.

**Fig 6:**
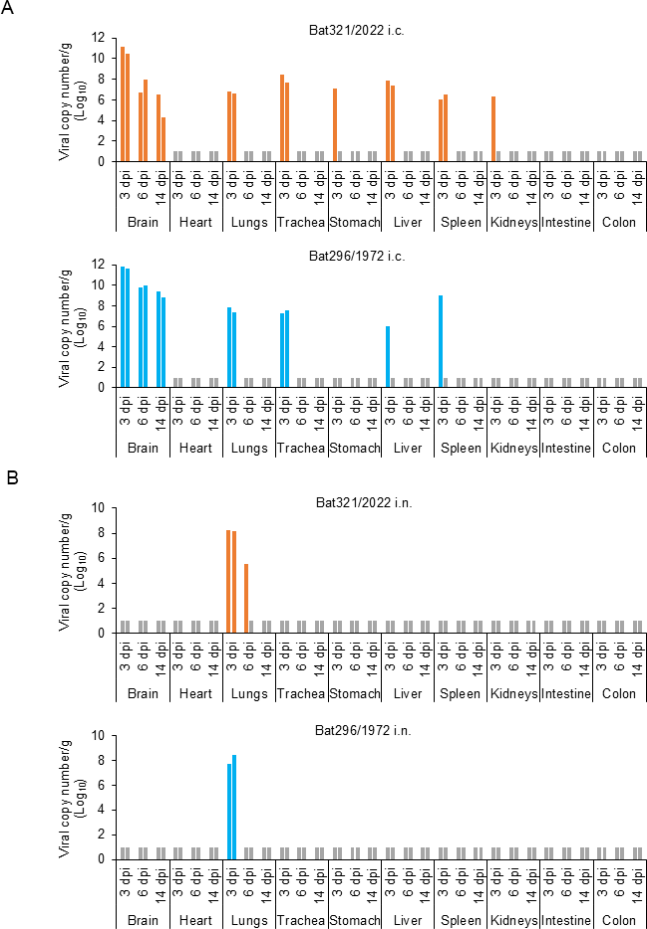
Viral gene copy numbers in the major organs of infected mice. Two mice per group were euthanized on 3, 6, and 14 dpi, and major organs (brain, heart, lungs, trachea, stomach, liver, spleen, kidneys, intestine, and colon) were collected. Viral copy numbers in homogenized organ samples were quantified by real-time RT-PCR (**A and B**). The gray bars indicate values below the detection limit.

To identify the sites of viral replication, viral gene copy numbers were measured in 10 organs using real-time RT-PCR (Fig. 6). High viral loads (>10^10^ copies/g) were detected in the brains of mice intracerebrally inoculated with either OITV 321/2022 or OITV 296/1972 on 3 dpi (Fig. 6A). In addition, viral RNA was detected in other organs, such as the lungs, trachea, liver, and spleen, on 3 dpi. Notably, a wide range of tissues, including the stomach and kidneys, tested positive for viral RNA in mice infected with OITV 321/2022. The brain viral load of OITV 321/2022 exceeded 10^8^ copies/g on 6 dpi and remained above 10^4^ copies/g on 14 dpi, whereas OITV 296/1972 maintained higher levels (10^8^ copies/g) on 6 and 14 dpi.

In mice inoculated intranasally with either OITV 321/2022 or OITV 296/1972, viral genes were detected only in the lungs on 3 dpi at levels exceeding 10^7^ copies/g (Fig. 6B). No viral genes were detected in the other organs, including the trachea. These results suggest that both OITV 321/2022 and OITV 296/1972 infected BALB/c mice via the intranasal route but remained largely localized and confined to the lungs and were cleared without systemic dissemination by 14 dpi.

## DISCUSSION

In the present study, two OITV strains were isolated from oral swab samples collected from *R. cornutus* in the Katano Cave, Kagoshima Prefecture, Japan (Table 1). Notably, this is the first isolation of OITV in 50 years since OITV 296/1972 was isolated from the blood of *R. cornutus* in the Oita Prefecture, Japan, in 1972 (16). Unlike previous blood-derived isolates, the Kagoshima isolates were recovered from oral swabs, suggesting viral shedding via this route. *R. cornutus* and other microbat species are widely distributed across Japan, from temperate to subarctic regions, and often form large colonies ranging from dozens to thousands of individuals (5). This broad geographical distribution suggests that OITV may circulate among these bat species throughout Japan and is not limited to the Kyushu region. Continued surveillance of OITV in bats across Japan will provide further insight into its epidemiology. Notably, none of the other viruses were detected from OITV-positive bats in this study, including bat sarbecoviruses, which have been isolated from the Katano Cave (17).

Among the viral genes, the P gene of OITV 321/2022 exhibited the lowest nucleotide identity (97.78%) with that of OITV 296/1972, whereas the other ORFs showed higher similarity (Table 2). Similar P gene variability has been reported for other rhabdoviruses, including rabies, Kanyawara, and Durham viruses (11, 18–20). Although the N gene is generally conserved among rhabdoviruses, a wide range of nucleotide identities (87.6%–100%) has been observed among N gene fragments of 60 RABV strains collected over a 2-year period in China (21). In contrast, the N genes of the Kagoshima isolates showed 99.15% nucleotide identity with OITV 296/1972, despite the 50-year gap, suggesting strong genetic conservation. It is known that viral mutation rates tend to be lower in insect cells than in mammalian cells (22), raising the possibility that OITV circulates primarily in arthropod hosts and is sporadically transmitted to microbats. OITV belongs to subgroup C of the genus *Ledantevirus*, members of which have been identified in both vertebrate and invertebrate hosts, including bat flies, supporting this hypothesis (Fig. 3) (10, 11, 23, 24).

Several viruses in the genus *Ledantevirus* have been shown to replicate in cells derived from a broad range of animal species. For example, a previous study reported that DPRV replicates in BHK, A549, and African green monkey kidney MA104 cells (6). Kumasi rhabdovirus replicates efficiently in Vero, MA104, A549, and kidney and lung cells from *Eidolon helvum* (EidNi/EidLu cells) (2), whereas the Kolente virus replicates in BHK cells (12). In this study, OITV 321/2022 could replicate in cell lines derived from various animal species, including multiple bat species (Fig. 4), but not in mosquito (*Aedes albopictus*; C6/36 cells) and tick (*Ixodes scapularis*; ISE6 cells) cell lines. These results suggest that OITV may circulate in other arthropod vectors, such as bat flies. Notably, OITV 321/2022 replicated in a human-derived A549 cell line, indicating a potential for human infection. Previous serological studies have shown that a substantial proportion of individuals have had prior exposure to ledanteviruses typically associated with mild or subclinical symptoms (2, 6). Further serological investigations are warranted to determine whether OITV infects humans in Japan.

OITV 296/1972 is known to replicate in the brains of BALB/c mice following intracerebral inoculation, causing lethal encephalitis in mice up to 2 weeks of age and inducing only transient signs, such as ruffled fur, in mice older than 4 weeks (16). In this study, intracerebral inoculation of BALB/c mice with OITV 321/2022 did not result in any clinical symptoms (Fig. 5A). However, a systemic infection was found on 3 dpi (Fig. 6A). Although OITV 321/2022 replicated in the brain, its viral titers declined over time, in contrast to OITV 296/1972, maintaining high viral loads in the brain throughout the 14-day experimental period (Fig. 6A).

A previous study reported that DPRV exhibited increased morbidity and mortality following serial intracerebral passages in mice (6). Similarly, OITV 296/1972 used in our study had been serially passaged and maintained in mouse brains, which may have enhanced its neurovirulence compared with the original field isolate. In contrast, OITV 321/2022 was directly inoculated into mice after isolation from cultured cells, thereby providing an assessment of its pathogenicity in a state likely closer to that in nature. Importantly, both OITV 321/2022 and OITV 296/1972 established infections in the lungs following intranasal inoculation (Fig. 5B and 6B; Fig. S2). Consistent with the virus isolation from oral swabs, these findings suggest that OITV may be transmitted among microbats via respiratory route.

In conclusion, OITV was isolated from the oral swabs of microbats in Kagoshima Prefecture, Japan, marking the first isolation of this virus since its original identification in 1972. Our findings demonstrate that OITV can infect and replicate in a broad range of animal cell lines, including those derived from humans, indicating a potential risk of cross-species transmission to domestic animals and humans. This study highlights the importance of continued surveillance and in-depth characterization of bat-associated viruses to better assess their potential for zoonotic transmission.

## MATERIALS AND METHODS

### Cells and medium

Cells derived from the kidneys of African green monkeys (Vero cells), Vero cells stably expressing angiotensin-converting enzyme 2 derived from *R. cornutus* (Vero-RcACE2 cells) (25), human adenocarcinoma cells derived from lung cancer A549 cells, and BHK cells were maintained in Dulbecco’s modified Eagle’s medium (DMEM) (Fujifilm Wako Pure Chemical Corporation, Osaka, Japan) supplemented with 10% fetal bovine serum (FBS), 10,000 U/mL penicillin, and 10 mg/mL streptomycin (PS) (Fujifilm Wako Pure Chemical Corporation) (10% FBS/DMEM). VeroE6 cells stably expressing human transmembrane serine protease 2 (VeroE6/TMPRSS2 cells) (26) were grown in 10% FBS/DMEM supplemented with 100 µg/mL G418 (InvivoGen, San Diego, CA, USA). The bat cell line, TB1 Lu (*Tadarida brasiliensis*, lung; ATCC number CCL-88), was maintained in Eagle’s minimum essential medium (EMEM) (Fujifilm Wako Pure Chemical Corporation) supplemented with 10% FBS and PS (10% FBS/EMEM). PK-15 cells were maintained in 10% FBS/EMEM supplemented with a non-essential amino acid solution (Nacalai Tesque, Kyoto, Japan). MDCK cells were maintained in MEM (Thermo Fisher Scientific) supplemented with 5% newborn calf serum, 0.9 mM sodium bicarbonate, MEM amino acids (Thermo Fisher Scientific), MEM vitamin solution (Fujifilm Wako Pure Chemical Corporation), 2 mM L-glutamine, and PS. After inoculation with swab samples or viral isolates, Vero, Vero-RcACE2, VeroE6/TMPRSS2, A549, and BHK cells were maintained in 2% FBS/DMEM, whereas TB1 Lu and PK-15 cells were maintained in 2% FBS/EMEM. Bat-derived cell lines, including DemKT1 (*Rousettus leschenaultii*, kidney), BKT1 (*R. ferrumequinum*, kidney), YubFKT1 (*Miniopterus fuliginosus*, kidney), IndFSPT1 (*Pteropus giganteus*, spleen), ZFBK 11-97 (*Epomophorus gambianus*, kidney), ZFBK 15-137RA (*Rousettus aegyptiacus*, kidney), and SuBK 12-08 (*M. schreibersii*, kidney) were cultured in Roswell Park Memorial Institute 1640 medium (Sigma–Aldrich) supplemented with 10% FBS and PS. All bat cell lines were maintained at 37°C in an atmosphere with 5% CO_2_ (27). The tick cell line ISE6, derived from embryonated eggs of *I. scapularis*, was cultured in L-15B medium containing 10% FBS and 5% tryptose phosphate broth (Sigma–Aldrich) at 32°C without CO_2_. The mosquito cell line C6/36, derived from *A. albopictus*, was maintained in MEM supplemented with 10% FBS, PS, and 1% MEM non-essential amino acids (Gibco) at 28°C in an atmosphere with 5% CO_2_.

### Sample collection

Oral swab samples were collected from microbats captured in Katano Cave, Kagoshima Prefecture, in 2022 (Fig. 1) as reported previously (17). Microbats were captured using nets and released immediately after oral swab collection. Species were identified based on morphology. Age class, adult or young, was determined according to the presence or absence of clear signs of sexual maturity. The swabs were suspended in laboratory-made transport medium (MEM supplemented with 0.5% bovine serum albumin, 2 mM L-glutamine, 0.3 mg/mL gentamicin, 2.5 µg/mL amphotericin B, and PS) and stored at 4°C or lower. The samples were immediately subjected to viral isolation on the day of collection.

### Virus isolation

Suspended swab samples were used for virus isolation (17, 28). In brief, the supernatant was filtered through a 0.2 µm pore membrane (Sartorius, Göttingen, Germany) and inoculated into Vero-RcACE2 cells. After an hour of incubation at 37°C, the inoculum was replaced with fresh medium. The samples were subjected to blind passage thrice at 1-week intervals. Supernatant from cells showing detachment was collected and stored at −80°C. Viral titers were assessed in each cell line using a median TCID_50_ assay.

### Transmission electron microscopy

Supernatant collected from cells infected with our isolate, OITV 321/2022, was filtered through a 0.2 µm pore membrane (Sartorius) and subjected to ultracentrifugation (Himac CS 120GX, Eppendorf Himac Technologies Co., Ltd., Ibaraki, Japan) at 24,000 rpm at 4°C for 2 h with a sucrose cushion (15% sucrose in PBS). The precipitate was fixed with 1% formaldehyde and stained negatively with phosphotungstic acid. The prepared samples were examined under an H7000KU electron microscope (Hitachi, Tokyo, Japan) operated at 80 kV.

### Full-genome sequencing

Nucleic acids were extracted from the precipitate obtained by ultracentrifugation using an innuPREP Virus DNA/RNA Kit (IST Innuscreen GmbH, Reinach, Switzerland). Double-stranded cDNA (dscDNA) was synthesized from the total RNA using the PrimeScript Double Strand cDNA Synthesis Kit (TaKaRa Bio, Kusatsu, Japan). Next, dscDNA was sequenced using a MinION Mk1B nanopore sequencer with a Flongle flow cell and a direct cDNA Sequencing Kit (SQK-DCS109) (Oxford Nanopore Technologies, Oxford, UK) or a reagent kit designed for sequencing (MiSeq Reagent Kit v3, Illumina, San Diego, CA, USA; 600 cycles). The obtained contigs were assembled *de novo* using the Geneious Prime software (Dottmatics, Boston, MA, USA). Consensus sequences were subjected to BLAST analysis (https://blast.ncbi.nlm.nih.gov).

### Phylogenetic analyses

Nucleotide sequences of *Rhabdoviridae* were retrieved from the NCBI nucleotide database (https://www.ncbi.nlm.nih.gov/nucleotide/). Sequences were aligned using the MUSCLE software (29). Phylogenetic analyses of the nucleotide sequences from our isolates and other Rhabdoviruses were conducted using IQ-TREE software (30) with bootstrap analyses of 1,000 replicates. The best-fit model (GTR + F + I + R7) was selected by IQ-TREE software.

### Real-time RT-PCR

Real-time RT-PCR was performed using an iTaq Universal SYBR Green One-Step Kit (Bio-Rad, Hercules, CA, USA) following the manufacturer’s protocol. Primer sets were designed based on the sequence of the OITV 321/2022 L gene to generate 163 bp PCR products (OITV 321/2022 1797F: ACAATGGCGGATGACAT and OITV 321/2022 1958R: CCCAGGAAAGCTCCCAT). The PCR product of the OITV 321/2022 L gene was cloned into a pCR Blunt II-TOPO cloning vector (Thermo Fisher Scientific).

### Evaluation of growth kinetics based on the virus titer

OITV 321/2022 was inoculated into Vero, Vero-RcACE2, and VeroE6/TMPRSS2 cells at a multiplicity of infection (MOI) of 0.001, while A549, BHK, and MDCK cells were infected at an MOI of 1.0 calculated in Vero-RcACE2 cells. Bat-derived, tick-derived, and mosquito-derived cells were inoculated with OITV 321/2022 at an MOI of 1.0. After an hour of incubation at 37°C, the inoculum was replaced with fresh medium. Supernatants were serially collected up to 168 hpi. The viral titer in each supernatant was determined using TCID_50_ assays in Vero-RcACE2 cells.

### Experimental infection in mice

Three-week-old female BALB/c mice were purchased from Japan SLC Inc. (Hamamatsu, Japan) and housed in animal biosafety level-2 facilities at the Joint Faculty of Veterinary Medicine, Kagoshima University, Japan. Overall, 34 mice were used in this study and inoculated intracerebrally (*n* = 7) or intranasally (*n* = 7) with OITV 321/2022, while the other group was inoculated intracerebrally (*n* = 7) or intranasally (*n* = 7) with OITV 296/1972. Negative controls were mice administered PBS intracerebrally (*n* = 3) or intranasally (*n* = 3). Mice were inoculated with OITV at 1.58 × 10^6^ TCID_50_ in 200 µL, determined using Vero-RcACE2 cells. Each mouse was monitored daily for clinical signs and weight loss until 14 dpi. Major organs (brain, heart, lungs, trachea, stomach, liver, spleen, kidneys, intestine, and colon) were collected from two mice from each OITV-inoculated group on 3, 6, and 14 dpi. Each collected organ was homogenized to prepare a 10% emulsion with a laboratory-made transport medium using a TissueLyser II (Qiagen, Hulsterweg, Netherlands). All organ homogenates were preserved at −80°C until nucleic acid extraction. RNA was extracted from organ homogenates using the MagMAX Viral/Pathogen Nucleic Acid Isolation Kit with KingFisher Duo Prime (Thermo Fisher Scientific) following the manufacturer’s instructions. The viral copy number was quantified for each specimen by real-time RT-PCR (see above). Whole blood was obtained from three mice from each OITV-inoculated group and negative control on 14 dpi. The whole blood samples were centrifuged at 10,000 rpm at 4°C for 5 min, and the serum was collected for subsequent microneutralization and ELISA. All procedures involving BALB/c mice were approved by the Institutional Animal Care and Use Committee of the Kagoshima University Experimental Animal Center (approval no. JFVM22049).

### Microneutralization assay

Serum samples collected from the BALB/c mice were serially diluted (10 × 2^0^ to 10 × 2^12^) and mixed with OITV 321/2022 or OITV 296/1972 at 240 TCID_50_. After 1 h of incubation, the serum-virus mixture was inoculated into Vero-RcACE2 cells in 96-well plates and cultured for 6 days. Wells showing CPE were scored as OITV-positive. The neutralizing antibody titer was defined as the highest serum dilution that completely prevented OITV infection.

### ELISA

Supernatants from Vero-RcACE2 cells infected with OITV 321/2022 and OITV 296/1972 were filtered through a 0.22 µm pore membrane (Sartorius). The viruses were concentrated via ultracentrifugation at 24,000 rpm in a Beckman SW32 Ti swinging-bucket rotor (Beckman Coulter) for 2 h with a sucrose cushion (15% sucrose in PBS). The resulting pellet was resuspended in disruption buffer containing 0.05 M Tris-HCl (pH 7.6), 0.5% Triton X-100, and 0.6 M KCl. Disrupted viral particles were diluted 1,000-fold in PBS and used as ELISA antigens. ELISA plates (Nunc Maxisorp; Invitrogen, Carlsbad, CA, USA) were coated with prepared viral antigens and blocked with 3% skim milk (Becton Biosciences, Franklin Lakes, NJ, USA) in PBS. Serum samples were initially diluted 1:50 in PBS containing 1% skim milk and 0.05% Tween 20, and then subjected to twofold serial dilutions up to 1:25,600. Each dilution was plated in triplicate. Bound antibodies were detected using a horseradish peroxidase-conjugated goat anti-mouse IgG antibody (H + L) (Abcam, Cambridge, UK). Antibody-antigen interactions were visualized using EzELISA TMB (ATTO Corporation, Tokyo, Japan). Optical density was measured at 450 nm to quantify the antibody response.

## Data Availability

All relevant data are within the article. The genome sequences of OITV 321/2022 and OITV 326/2022 were deposited in GenBank (https://www.ncbi.nlm.nih.gov/genbank/) under accession numbers PX116046 and PX116047.

## SUPPLEMENTAL MATERIAL

Supplemental material spectrum.03158-25-s0001.pdf Fig. S1 and S2.

ASM does not own the copyrights to Supplemental Material that may be linked to, or accessed through, an article. The authors have granted ASM a non-exclusive, world-wide license to publish the Supplemental Material files. Please contact the corresponding author directly for reuse.
