# Supplementary material for: Oita virus rediscovered after 50 years: isolation of genetically conserved strains from bats in Southern Japan
Source: Microbiol Spectr. 2025 Nov 11;13(12):e03158-25. doi: 10.1128/spectrum.03158-25 (PMC12671071; doi:10.1128/spectrum.03158-25)
Supplement: Supplemental material — Fig. S1 and S2. [file spectrum.03158-25-s0001.pdf]

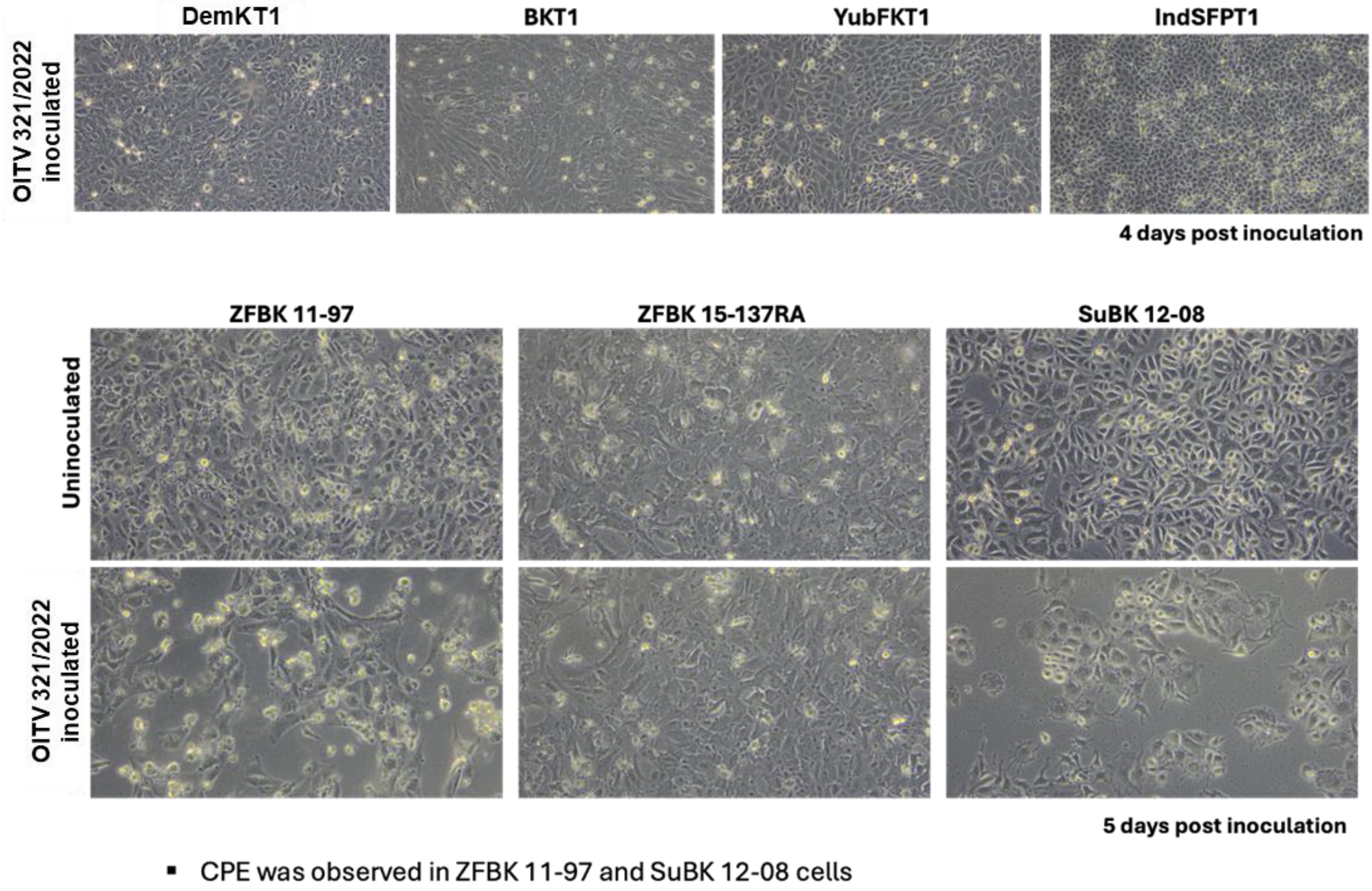

Supplementary Fig. S1. Bat-derived cell lines inoculated with OITV 321/2022.

Bat-derived cell lines were inoculated with OITV 321/2022 at a multiplicity of infection (MOI) of 1.0. Representative images show CPE observed at four or five days post-inoculation.

Fig. S1

A

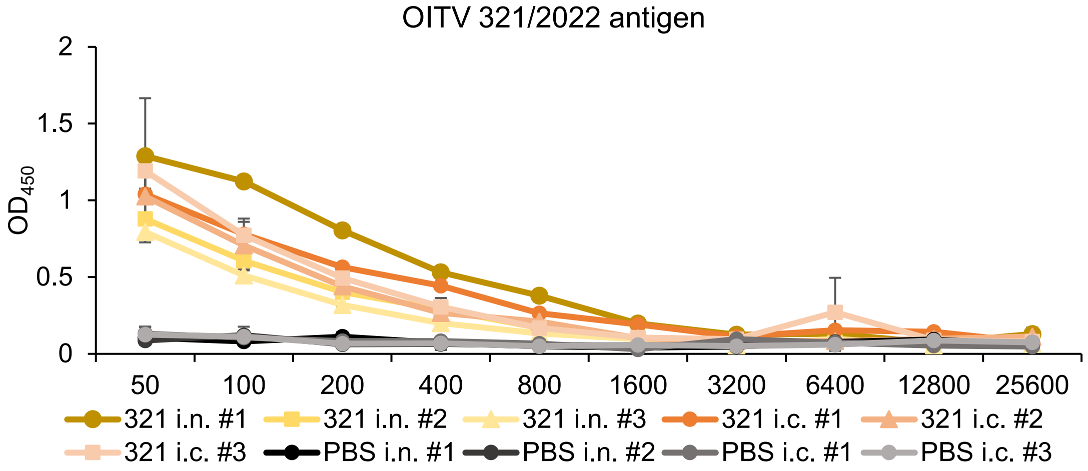

B

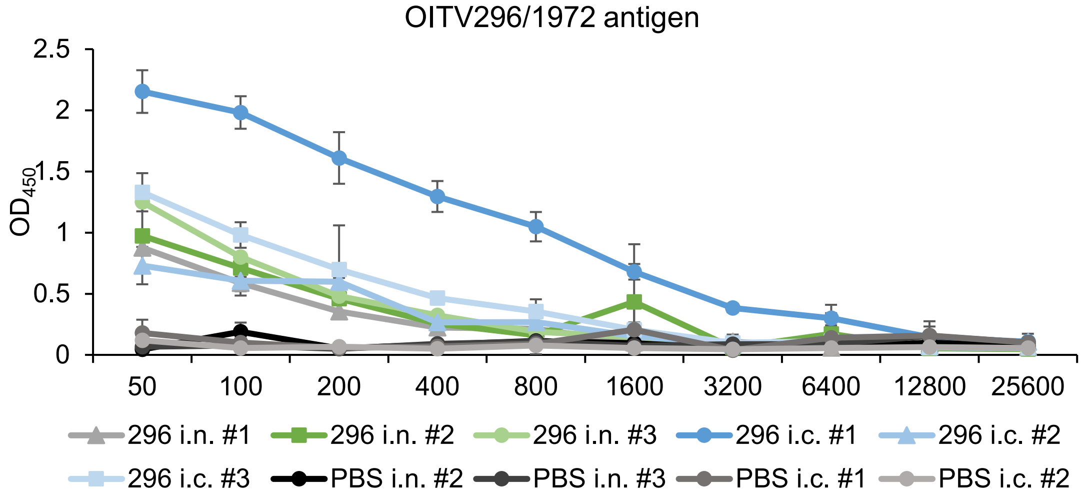

Supplementary Fig. S2. Detection of OITV-specific IgG antibodies in serum samples collected from mice. IgG antibodies reactive to the homologous whole virus antigens of OITV 321/2022 (A) and OITV 296/1972 (B) were measured by ELISA. Serum samples were initially diluted 1:50 and then subjected to two-fold serial dilutions up to 1:25,600 to determine antibody titers.

Fig. S2
